# Quantification of Uncertainty and Best Practice in Computing Interfacial Curvature from Complex Pore Space Images

**DOI:** 10.3390/ma12132138

**Published:** 2019-07-03

**Authors:** Takashi Akai, Qingyang Lin, Abdulla Alhosani, Branko Bijeljic, Martin J. Blunt

**Affiliations:** Department of Earth Science & Engineering, Imperial College London, London SW7 2AZ, UK

**Keywords:** pore-scale imaging, multiphase flow, capillary pressure, interfacial curvature, direct numerical simulation

## Abstract

Recent advances in high-resolution three-dimensional X-ray CT imaging have made it possible to visualize fluid configurations during multiphase displacement at the pore-scale. However, there is an inherited difficulty in image-based curvature measurements: the use of voxelized image data may introduce significant error, which has not—to date—been quantified. To find the best method to compute curvature from micro-CT images and quantify the likely error, we performed drainage and imbibition direct numerical simulations for an oil/water system on a bead pack and a Bentheimer sandstone. From the simulations, local fluid configurations and fluid pressures were obtained. We then investigated methods to compute curvature on the oil/water interface. The interface was defined in two ways; in one case the simulated interface with a sub-resolution smoothness was used, while the other was a smoothed interface extracted from synthetic segmented data based on the simulated phase distribution. The curvature computed on these surfaces was compared with that obtained from the simulated capillary pressure, which does not depend on the explicit consideration of the shape of the interface. As distinguished from previous studies which compared an average or peak curvature with the value derived from the measured macroscopic capillary pressure, our approach can also be used to study the pore-by-pore variation. This paper suggests the best method to compute curvature on images with a quantification of likely errors: local capillary pressures for each pore can be estimated to within 30% if the average radius of curvature is more than 6 times the image resolution, while the average capillary pressure can also be estimated to within 11% if the average radius of curvature is more than 10 times the image resolution.

## 1. Introduction

Immiscible fluid displacement in porous media is common in a variety of industrial applications, such as hydrocarbon recovery through water injection, carbon capture and storage in the subsurface (CCS) and biological water filtration [1,2,3]. When displacements occur, the arrangement of fluid phases in contact with each other and a solid is governed by energy balance; displacements move toward local energy minima at a pore-by-pore level [4]. Hence, it is of interest to study the interfaces of the phases since the change in the interfacial energy primarily controls the change in the energy of the system [5]. Furthermore, the curvature of the interface can be used to estimate capillary pressure from the Young-Laplace equation [6,7].

To help frame the analysis of interfaces, it is useful to introduce some properties of the surface. In general, at an arbitrary point on surface, two principal curvatures ($\kappa_{1}$ and $\kappa_{2}$) which are orthogonal to each other can be defined. Using these principal curvatures, mean curvature ($\kappa_{m}$) and Gaussian curvature ($\kappa_{G}$) are defined as:

(1) $$\begin{array}{ccc} \kappa_{m} & = & \frac{1}{2}\left(\kappa_{1}+\kappa_{2}\right), \end{array}$$

(2) $$\begin{array}{ccc} \kappa_{G} & = & \kappa_{1}\kappa_{2}. \end{array}$$

There are other useful measures of the topology of surface called Minkowski functionals. The zero-order Minkowski functional ($M_{0}$) is the volume enclosed by a surface. The first- to third-order Minkowski functionals are defined as:

(3) $$\begin{array}{ccc} M_{1} & = & \int dS, \end{array}$$

(4) $$\begin{array}{ccc} M_{2} & = & \int \kappa_{m}dS, \end{array}$$

(5) $$\begin{array}{ccc} M_{3} & = & \int \kappa_{G}dS. \end{array}$$

The first-order Minkowski functional ($M_{1}$) is the total area of the surface, the second-order ($M_{2}$) and the third-order Minkowski functional ($M_{3}$) are the surface integral of mean curvature and Gaussian curvature, respectively.

In immiscible fluid displacements, based on the Young-Laplace equation, capillary pressure ($P_{c}$) which is the pressure difference across the interface locally is related to the mean curvature by:

(6) $$\begin{array}{c} P_{c}=2\sigma \kappa_{m}, \end{array}$$

where σ is the interfacial tension between the two phases. Traditionally, in oil/water systems, the capillary pressure is measured in a laboratory based on the macroscopic definition of $P_{c}=P_{o}-P_{w}$, where $P_{o}$ and $P_{w}$ are the pressure in oil and water phases, using, for instance, the porous plate method in which the pressure of each phase is measured using two external pressure transducers [8,9].

More recently, high-resolution three-dimensional X-ray imaging has been used to measure the curvature of the interface extracted from segmented CT images during two-phase flow experiments to derive capillary pressure using Equation (6). Armstrong et al. [6] used synchrotron-based tomographic datasets of oil/water drainage and imbibition experiments on a bead pack [10]. The capillary pressure obtained from curvature measurements showed fairly good agreement with that obtained from pressure transducers for imbibition cycles, whereas a systematically lower capillary pressure was estimated for drainage. Li et al. [11], applying their improved curvature computation method to the same dataset, provided a better agreement between the pressure measurements and values derived from curvature computation. Using a similar curvature measurement method, Herring et al. [12] estimated the capillary pressure for a range of non-dimensional curvature between 0 and approximately 0.225 voxel${}^{-1}$ based on their air/water drainage and imbibition experiments on a Bentheimer sandstone.

The use of image based interface curvature measurements is not limited to the estimation of capillary pressure. AlRatrout et al. [13] showed the relationship between the degree of wettability alteration and pore wall surface roughness. Using micro-CT images obtained during water-flooding experiments on altered wettability rocks, they performed curvature measurements on both the oil/water interface and the pore wall surface while providing an estimate of contact angle at three-phase contact lines. They demonstrated a relationship between surface roughness, curvature and contact angle, where rougher surfaces, which retained water after primary drainage, tended to be more water-wet. Later, Lin et al. [14,15] demonstrated the distinct difference in flow behavior between a water-wet and mixed-wet state based on water-flooding experiments on Bentheimer sandstones. They observed that for the mixed-wet system the oil/water interfaces had two equal, but opposite, curvatures in orthogonal directions which means that they are approximately minimal surfaces, resulting in efficient displacement of oil. There is a distinct difference in the Gaussian curvature—the product of the two principal curvatures—with wettability. Strongly water-wet systems have interfaces with a positive Gaussian curvature, while mixed-wet media have mainly interfaces with a negative Gaussian curvature. These experimental findings can be interpreted using the Gauss-Bonnet theorem [16]:

(7) $$\begin{array}{c} \chi =\frac{1}{4\pi }M_{3}, \end{array}$$

where χ is the Euler characteristic which is the number of oil clusters minus the number of holes in the clusters. A smaller, negative, χ is associated with better connectivity. $M_{3}$ is the third-order Minkowski functional (see Equation (4)) computed on both the oil/water and oil/solid interfaces. This relation suggests that the negative Gaussian curvature observed in mixed-wet states is related to a negative χ, hence better phase connectivity.

Using the same experimental datasets as in Lin et al. [14,15], Blunt et al. [17] used the measured interface curvatures to derive a thermodynamically consistent contact angle based on conservation of the Helmholtz free energy. This methodology uses curvature and interfacial area measurements directly to inform the contact angle to be used in pore-scale modeling studies. Khanamiri et al. [18] provided a geometric description of the free energy of a porous system with two immiscible fluids using a linear combination of Minkowski functionals. Combining the geometric description with a thermodynamic description of free energy [5], they have estimated the amount of dissipated energy in drainage and imbibition processes.

However, there is an inherited difficulty in these image-based curvature measurements: the use of voxelized images may introduce significant error, which has not—to date—been quantified. Although several studies have discussed the validity of the method based on comparison between macroscopic capillary pressure obtained from transducers and the average or peak value of the computed mean curvatures, a wide range with some negative values have been observed, which are not expected for the water-wet media studied [6,11,12]. In fact, it is not clear how the distribution of measured curvatures represents the true local capillary pressure variation, which should be negligible in capillary equilibrium.

We study the accuracy of the image-based curvature computation using direct numerical simulations of drainage and imbibition for an oil/water system in complex porous media. Color-gradient two-phase lattice Boltzmann simulations are performed on synthetic images of a bead pack and micro-CT images of a Bentheimer sandstone, then the fluid configuration and pressure distribution after drainage and imbibition are obtained. We use these computations as a benchmark against which to compare image-based estimates of curvature. We show the comparison between computed curvatures on oil/water interface surfaces and those obtained from fluid pressure calculating both an averaged value for an entire sample and the pore-by-pore distribution. We show that the wide range of computed curvature distribution in a simple water-wet state in previous studies is likely due to errors in the measurements, and then suggest the best method to compute curvature from images and quantify the likely error.

## 2. Methods

### 2.1. The Color-Gradient Two-Phase Lattice Boltzmann Method

The color-gradient LB model proposed by Halliday et al. [19] was used. Our LB model was constructed with a 3D19Q lattice model which consists of a set of 19 discrete lattice velocity vectors, $e_{i}$ in three-dimensional space. We define particle distributions of two immiscible fluids, labeled red and blue, as $f_{i}^{r}$ and $f_{i}^{b}$, respectively. The fluid density ($\rho_{r}$ and $\rho_{b}$) and velocity (u) at position x and time *t* are obtained by:

(8) $$\begin{array}{ccc} \rho_{k} & = & \sum_{i}f_{i}^{k}\left(x,t\right),\;k\;=\;r\;\mathrm{or}\;b, \end{array}$$

(9) $$\begin{array}{ccc} \rho u & = & \sum_{i}f_{i}\left(x,t\right)e_{i}, \end{array}$$

where $f_{i}$ is the total particle distribution given by $f_{i}=f_{i}^{r}+f_{i}^{b}$; ρ is the total fluid density given by $\rho =\rho_{r}+\rho_{b}$. The lattice Boltzmann equation for the total particle distribution is written as:

(10) $$\begin{array}{c} f_{i}\left(x+e_{i}\delta t,t+\delta t\right)=f_{i}\left(x,t\right)+\Omega_{i}\left(x,t\right)+\phi_{i}, \end{array}$$

where δt denotes the lattice time step which is set to unity and $\Omega_{i}\left(x,t\right)$ and $\phi_{i}$ are the collision operator and the body force term, respectively. For the collision operator, we used the Multiple-Relaxation-Time (MRT) collision operator [20] expressed as:

(11) $$\begin{array}{c} \Omega_{i}=-{\left(M^{-1}SM\right)}_{i,j}\left[f_{j}-f_{j}^{eq}\right], \end{array}$$

where M and S are the transformation matrix and the diagonal relaxation matrix, respectively. $f_{i}^{eq}$ is the equilibrium distribution function which is obtained by a second-order Taylor expansion of the Maxwell-Boltzmann distribution with respect to the local fluid velocity. The location of the interface was tracked using a color function $\rho^{N}$ defined by:

(12) $$\begin{array}{c} \rho^{N}\left(x,t\right)=\frac{\rho_{r}\left(x,t\right)-\rho_{b}\left(x,t\right)}{\rho_{r}\left(x,t\right)+\rho_{b}\left(x,t\right)},\;-1\leq \rho^{N}\leq 1. \end{array}$$

Using the color function, the interfacial tension between two fluids was computed as a spatially varying body force, F, based on the continuum surface force (CSF) model [21] given by:

(13) $$\begin{array}{c} F=-\frac{1}{2}\sigma \kappa_{m}\nabla \rho^{N}, \end{array}$$

where σ is the interfacial tension and $\kappa_{m}$ is the mean curvature of the interface. This spatially varying body force F is incorporated into the lattice Boltzmann equation through the body force term $\phi_{i}$. For the MRT model, this is performed by transforming the forcing term proposed by Guo et al. [22] using the scheme presented in Yu and Fan [23]. After application of the interfacial tension (F) to the particle distributions, the recoloring algorithm proposed by Latva-Kokko and Rothman [24] is applied to these distributions to ensure phase segregation and maintain the interface. This results in a slightly diffusive interface whose thickness is about 2 to 3 lattice units. Further details of our LB model are provided in our previous publications [25,26]. The only difference is that we used the MRT collision operator, while the Single-Relaxation-Time (SRT) collision operator [27] was used in [25,26].

At solid-fluid boundary lattice nodes, a full-way bounce back boundary condition was implemented to achieve the non-slip boundary condition. In addition, the wettability of the solid surface was modeled by specifying contact angles using the wetting boundary condition presented in Akai et al. [25]. This wetting boundary condition accurately assigns contact angles for 3D arbitrary geometries with smaller spurious currents compared to the widely used fictitious density boundary condition [25]. For the inlet and outlet boundaries of a simulation domain, we used a constant pressure and velocity boundary condition [28].

### 2.2. Curvature Computation on Voxelized Images

We used a curvature computation method presented in the literature [6,7,11,12,14,29]. In this approach, curvature was measured on a smoothed fluid interface extracted from voxelized, segmented image data through the fitting of a quadratic form locally to the interface [29].

We started with three-phase segmented label data (oil, water and solid) obtained from raw gray-scale CT images. Using the marching cubes algorithm [30], the oil/water interface was extracted from the label data. Since this surface had a staircase shape, it had to be smoothed before computing curvature. In this study, we used Laplacian smoothing [31] since our previous study [32] showed that Laplacian smoothing gave the most accurate curvature estimation among the three smoothing methods tested: constrained Gaussian smoothing, Laplacian smoothing and boundary preserving Gaussian smoothing [33].

The extracted surface with a staircase shape was modeled as a triangulated surface, then Laplacian smoothing, in which the position of a vertex is moved to the average position of its neighboring vertices in a single iteration, was applied with multiple iterations on the surface. In this study, the extraction of the interface with the marching cubes algorithm was performed with commercial image analysis software, while Laplacian smoothing was performed with Paraview.

After the generation of a smoothed triangulated surface, the elemental triangles were fitted by a quadratic form:

(14) $$\begin{array}{c} ax^{2}+by^{2}+cz^{2}+2exy+2fyz+2gzx+2lx+2my+2nz+d=0. \end{array}$$

Then, the principal curvatures and directions of the principal curvatures were obtained from the eigenvalues and eigenvectors of Equation (14) [6]. The number of neighboring triangles to be used for the fitting at the center of a triangle can be chosen. We used a fixed value of 4 neighbors in the following analyses.

## 3. Results

First, in Section 3.1, using analytical surfaces, the validity of the curvature computation method described in Section 2.2 was investigated. Based on the analytical surfaces, synthetic voxelized data were generated. Curvature was estimated on both the analytically generated surfaces and the surfaces extracted from the synthetic voxelized data. The accuracy of the curvature computation was evaluated by comparing computed curvature values with the analytical values. Second, in Section 3.2, to demonstrate the error in curvature computation near three-phase contact lines, the oil/water interface during a drainage event in a square tube was generated by direct numerical simulation, then computed curvature values on the interface were compared with analytical values. Finally, in Section 3.3, we measure curvature on the oil/water interface after drainage and imbibition processes using the results of direct numerical simulations performed on synthetic images of a bead pack and micro-CT images of a Bentheimer sandstone.

### 3.1. Curvature of Analytical Surfaces

We studied a sphere and a catenoid. The former represents the oil/water interface found in a two-phase displacement process for a strongly water-wet or a strongly oil-wet medium, while the latter represents the interface observed for a mixed-wet state after aging of rock samples whose local contact angles varies around 90${}^{\circ }$ [15,17]. A catenoid has a zero mean curvature, with two equal and opposite curvatures in orthogonal directions. Hence, the Gaussian curvature is negative [34].

**Figure 1:**
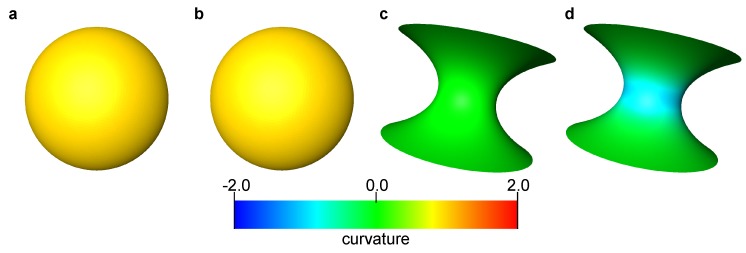
Analytical surfaces of the sphere (**a**,**b**) and catenoid (**c**,**d**). The surfaces are colored by the computed mean curvatures (**a**,**c**) and Gaussian curvatures (**c**,**d**). The analytical solutions are provided in Equation (A3).

**Figure 2:**
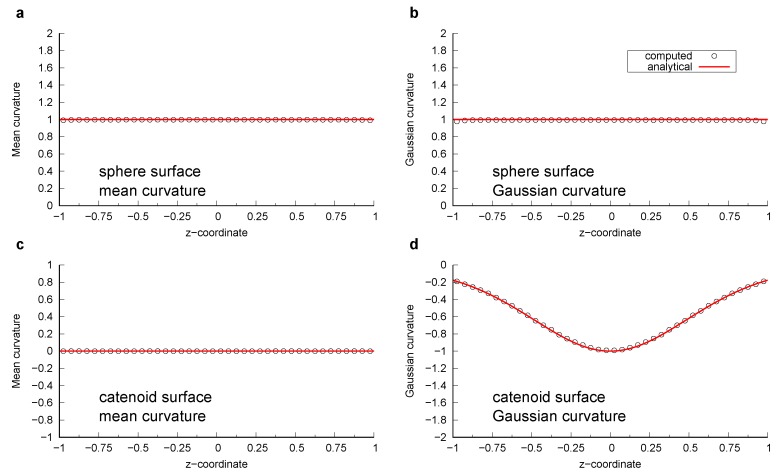
Comparison between computed and analytically obtained mean and Gaussian curvature as a function of *z*-coordinate. (**a**) Mean curvature of the sphere; (**b**) Gaussian curvature of the sphere; (**c**) Mean curvature of the catenoid; (**d**) Gaussian curvature of the catenoid.

The sphere had a unit radius, while the minimum radius of curvature of the catenoid was also 1 (see Appendix A, Equation (A1)). 201 × 101 data points were generated by changing the parameters *u* and *v* for every π/100 in the range (u,v)∈[0,2π]×[−1/2π,1/2π]. A surface was generated that was comprised of triangles connecting analytically computed points. We refer to these surfaces as an analytical surface. The curvature was computed on the analytical surfaces using the method described in Section 2.2. Figure 1 shows these analytical surfaces colored by computed local mean and Gaussian curvatures. The computed curvatures were sampled for every 0.05 size interval in the *z*-direction within the range z∈[−1:1], then average values of the mean and Gaussian curvature were calculated for each interval and compared with their analytical values (Figure 2). For all bins, the standard deviation of the computed mean and Gaussian curvatures was of the order of 10${}^{-3}$. This suggests that when the surface is sufficiently smooth, the standard method to compute curvature in which a quadratic equation is fitted to surfaces provides an accurate estimation with negligible standard deviation.

Next, synthetic voxelized data were generated based on the analytical surfaces. The rectangular region (x,y,z)∈[−1.5,1.5]×[−1.5,1.5]×[−1,1] was sampled with 4 grid sizes of Δ = 0.025, 0.05, 0.1 and 0.2. Then, a binary label was assigned to each grid block based on the analytical surfaces, i.e., a grid block was labeled as 0 for the inside of the analytical surface and labeled as 1 for the outside. The interface between these labels, which had a staircase shape, was extracted from the synthetic voxelized data. Then, applying Laplacian smoothing, smooth surfaces were generated. We refer to these surfaces as a smoothed surface. Consequently, 4 smoothed surfaces corresponding to grid sizes of Δ = 0.025, 0.05, 0.1 and 0.2 were prepared for the sphere and catenoid.

**Figure 3:**
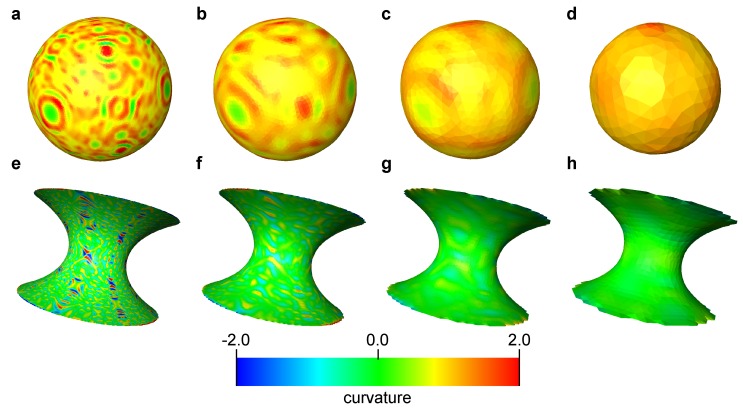
The smoothed surfaces extracted from the voxelized images for different grid sizes. The sphere surface with (**a**) Δ=0.025, (**b**) Δ=0.05, (**c**) Δ=0.1 and (**d**) Δ=0.2. The catenoid surface with (**e**) Δ=0.025, (**f**) Δ=0.05, (**g**) Δ=0.1 and (**h**) Δ=0.2. These surfaces were obtained with 600 iterations of Laplacian smoothing and are colored by the computed mean curvature.

**Figure 4:**
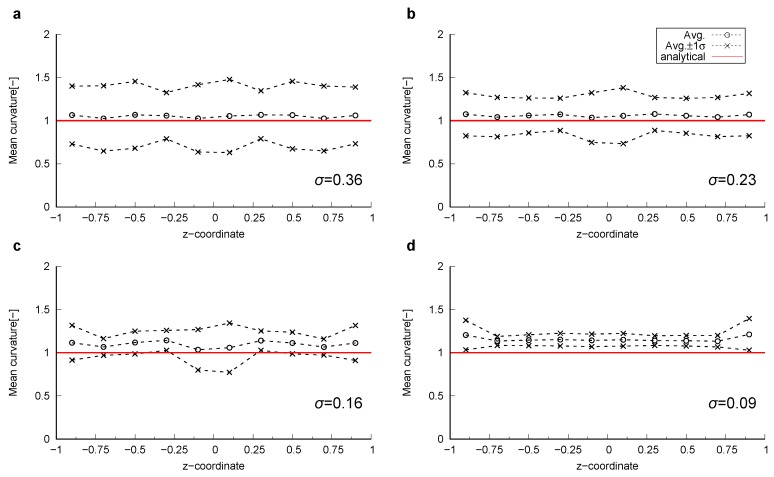
The computed mean curvatures together with the values plus and minus one standard deviation (σ) on the smoothed surfaces of the sphere compared to the analytical value as a function of the *z*-coordinate. The mean curvature of the sphere for (**a**) Δ=0.025, (**b**) Δ=0.05, (**c**) Δ=0.1 and (**d**) Δ=0.2.

**Figure 5:**
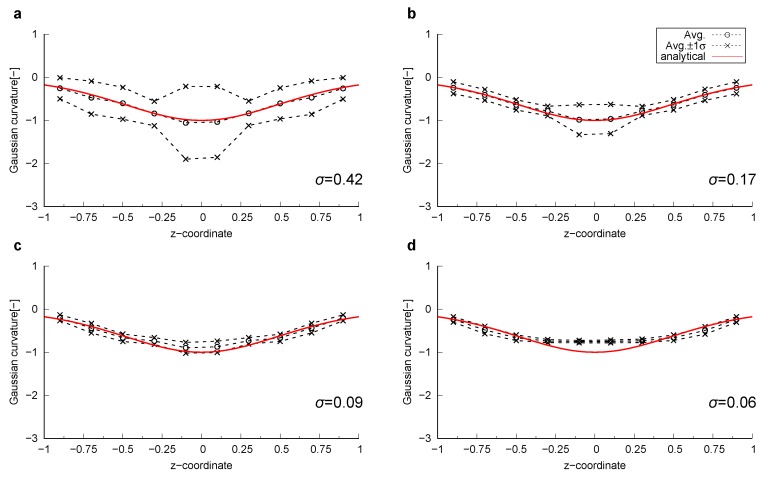
The computed Gaussian curvatures together with the values plus and minus one standard deviation (σ) on the smoothed surfaces of the catenoid compared to the analytical value as a function of the *z*-coordinate. The Gaussian curvature of the catenoid for (**a**) Δ=0.025, (**b**) Δ=0.05, (**c**) Δ=0.1 and (**d**) Δ=0.2.

Curvature was computed on the smoothed surfaces. Figure 3 shows these smoothed surfaces colored by the computed local mean curvatures. Here, the surfaces were obtained using 600 smoothing iterations. The impact of the number of smoothing iterations will be discussed later in this section. The computed local curvatures were sampled for every 0.2 size interval, which is the largest grid block size among the 4 grid sizes, in the *z*-direction within the range z∈ [−1:1]. Then, the average and standard deviation of the mean and Gaussian curvature for each interval were computed. The comparison between computed and analytical values for mean curvature of the sphere and Gaussian curvature of the catenoid are shown in Figure 4 and Figure 5, respectively. As shown in Figure 4d and Figure 5d, when the grid size does not have sufficient resolution to capture a feature, using too many smoothing iterations causes shrinkage of a feature in the sphere and flattening of a feature in the catenoid around z=0, resulting in errors in the estimation of curvature.

**Figure 6:**
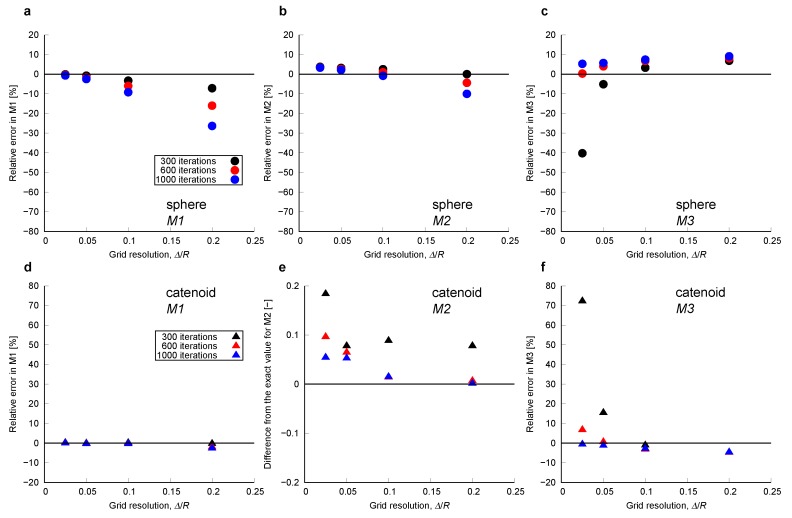
Errors in the estimation of $M_{1}$, $M_{2}$ and $M_{3}$ as a function of dimensionless resolution, Δ/R. $M_{1}$ is the surface area, $M_{2}$ is the surface-averaged mean curvature and $M_{3}$ is the surface-averaged Gaussian curvature. Errors in (**a**) $M_{1}$, (**b**) $M_{2}$ and (**c**) $M_{3}$ for the sphere. Errors in (**d**) $M_{1}$, (**e**) $M_{2}$ and (**f**) $M_{3}$ for the catenoid. Different iterations of the smoothing procedure were considered.

To quantify error in relation to the grid resolution of the original voxelized data, we define a dimensionless feature size, $\kappa^{*}=\Delta /R$, where *R* is the minimum absolute value of the radius of curvature, which is unity for our sphere and catenoid. The first- to third-order Minkovski functionals, $M_{1}$, $M_{2}$ and $M_{3}$, of the sphere and catenoid were obtained from the computed curvature on the smoothed surfaces. These functionals quantify the surface area, and the surface-averaged mean and Gaussian curvature—see Appendix A, Equations (A4) and (A5) for the mathematical details. Then, they were compared with their analytical values obtained with Equations (A4) and (A5). Here, we also examined the impact of smoothing by changing the number of smoothing iterations. Figure 6 shows the errors in the estimation of $M_{1}$, $M_{2}$ and $M_{3}$. As we can see in Figure 6a,b for $\kappa^{*}=0.2$ with 600 and 1000 iterations: over-smoothing causes shrinkage in an object, resulting in an underestimate of $M_{1}$, the surface area, with an overestimate of curvature. In these cases, the errors cancel in the calculation of $M_{2}$, since we integrate a larger curvature over a smaller area. In contrast, when the dimensionless feature size is large, insufficient smoothing can cause significant errors in the estimation of $M_{3}$ as seen in Figure 6c,f for $\kappa^{*}=0.025$ with 300 iterations.

There was an optimum resolution to accurately compute curvature with voxelized data. When the resolution of original voxelized data was too fine, computed curvatures showed a large standard deviation. This large standard deviation could be reduced by applying more smoothing by increasing the number of smoothing iterations. On the other hand, when the resolution was coarse, there was a significant deviation from the analytical solution. This deviation was caused by the shrinkage of the sphere and by the flattening of the catenoid. Overall, the curvature computation method described in Section 2.2 with 600 iterations of Laplacian smoothing estimated curvature within a 10 % error for the range of dimensionless feature size $0.025\leq \kappa^{*}\leq 0.2$.

### 3.2. Curvature of the Oil/Water Interface in a Simple Pore Geometry

#### 3.2.1. Simulation Conditions

In this section we estimate interfacial curvature where a solid is present. Drainage simulations for a square tube with a pore width of 36 μm whose inscribed pore radius is therefore 18 μm were performed. A simulation domain consisting of 144 × 36 × 36 grid blocks with a resolution of 1 μm/voxel was used. Void buffer regions of 12 × 60 × 60 grid blocks were attached to the inlet and outlet of the simulation domain. Oil, the non-wetting phase, was injected in the *x* direction from the inlet face at *x* = 0 with a constant velocity, while a constant pressure boundary condition was applied for water, the wetting phase, at the outlet face at *x* = 168 μm. The capillary number was defined as $Ca=\mu_{o}q_{o}/\sigma$, where $\mu_{o}$ and $q_{o}$ are the viscosity and Darcy velocity (the total flow rate divided by the cross-sectional area perpendicular to the flow direction) of the injected oil and σ is the interfacial tension between the oil and water. The injection velocity of oil was chosen to achieve a capillary number of $Ca=1\times 10^{-5}$, which is sufficiently low that viscosity forces were insignificant. Six simulations were conducted with different contact angles ranging from 0${}^{\circ }$ to 75${}^{\circ }$ to have a range of radius of curvature of the interface. The simulations were stopped when the interface reached the middle of the domain.

#### 3.2.2. Curvature of the Interface

First, to validate our direct numerical simulation model, the capillary pressure ($P_{c}^{sim}$) was obtained from the simulated fluid pressure in the oil and water phases, based on the following:

(15) $$\begin{array}{c} P_{c}^{sim}=\frac{1}{N_{o}}\sum_{\phi >0.9}P^{sim}-\frac{1}{N_{w}}\sum_{\phi <-0.9}P^{sim}, \end{array}$$

where $N_{o}$ and $N_{w}$ are the number of oil and water voxels whose color function is $\rho^{N}$ > 0.9 and $\rho^{N}$ < −0.9, respectively (see Equation (12) for the definition of the color function). $P^{sim}$ is the fluid pressure in each voxel. Here, we used the voxels with $|\rho^{N}|>0.9$ to remove oil/water interface regions in the simulations. The capillary pressure obtained was compared with the analytically derived capillary pressure provided in Øren et al. [35] for which wetting layers were present in the corners ($\theta <45^{\circ }$) and in Blunt [4] for which wetting layers were absent ($\theta \geq 45^{\circ }$). As shown in Table 1, the relative error in the estimation of capillary pressure was less than 5.6% for the range of contact angles studied ($0^{\circ }\leq \theta \leq 75^{\circ }$). This study demonstrates that for a simulation study we have an independent method to compute capillary pressure and curvature, which does not depend on the explicit consideration of the shape of the fluid-fluid interface.

**Figure 7:**
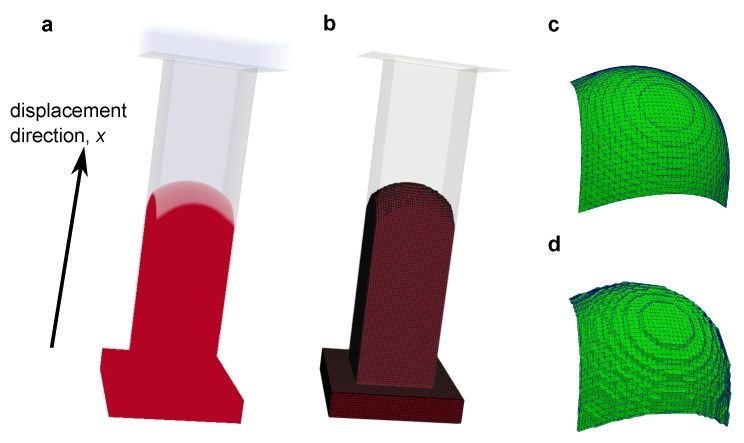
An example of the simulation results for a contact angle of $\theta =45^{\circ }$. (**a**) The simulated phase distribution. Oil is shown in red, while water and solid phase are transparent; (**b**) The synthetic voxelized data from the simulated phase distribution. Here, only oil is shown; (**c**) The simulated interface extracted from the simulated phase distribution corresponding to the color function of $\rho^{N}=0$ (see Equation (12)) (**d**) The staircase interface extracted from the synthetic voxelized data.

**Figure 8:**
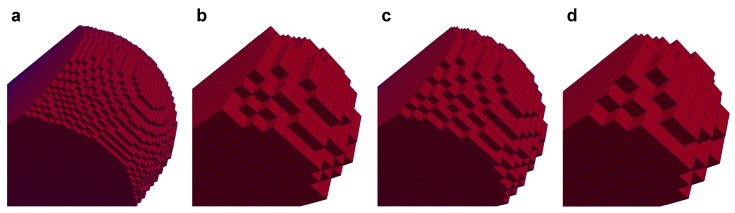
Examples of the synthetic voxelized data resampled from the simulated phase distribution for a contact angle of $\theta =45^{\circ }$ for grid sizes of (**a**) Δ = 1, (**b**) 2, (**c**) 3 and (**d**) 4 μm.

Next, two types of surface were prepared for the computation of curvature. It is possible to extract the oil/water interface from the simulations with a sub-grid resolution smoothness. This was performed by extracting the surface corresponding to the color function $\rho^{N}=0$. We refer to these surfaces as simulated interfaces. An example of our simulated phase distribution and the simulated interface is shown in Figure 7. The other surface is a smoothed interface. This surface was generated based on synthetic voxelized data from the simulated phase distribution. First, simulation results with a resolution of 1 μm were labeled as oil, water and solid based on the value of color function in a grid block (Figure 7c). This synthetic voxel data was then resampled to different grid sizes of Δ = 1, 2, 3 and 4 μm as shown in Figure 8. Then, the staircase interface extracted from the label data (Figure 7d) was smoothed with 600 iterations of Laplacian smoothing.

**Figure 9:**
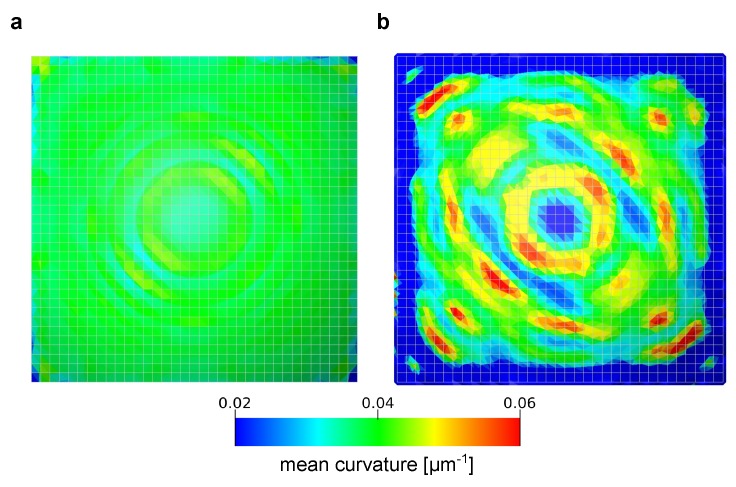
Distribution of computed mean curvature on (**a**) the simulated surface and (**b**) the smoothed surface for $\theta =45^{\circ }$. The grid size of these surfaces are 1 μm/voxel. The analytical value of mean curvature for this case is $\kappa_{m}$ = 0.04 μm${}^{-1}$ and uniform everywhere. We see large errors near the three-phase contact line.

**Figure 10:**
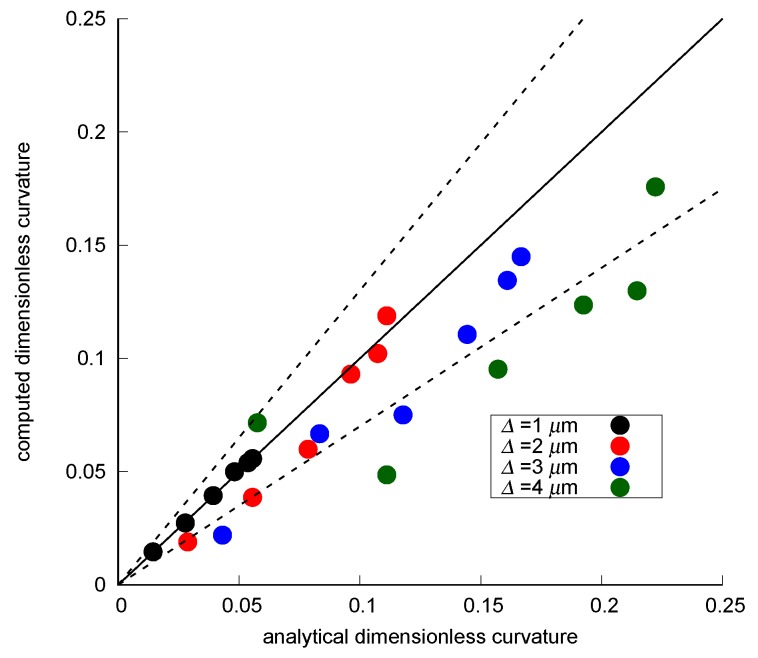
Comparison between analytical and computed dimensionless curvature ($\kappa^{*}$) for the 24 surfaces. ±30% error is indicated by the dotted lines.

The curvature was then computed on total of 24 smoothed interfaces (6 contact angles × 4 grid resolutions). Since significant error was observed near the three-phase contact lines in the smoothed interfaces as shown in Figure 9, we decided to discard curvature values within 3 voxels from the pore walls. Figure 10 shows the comparison between analytical and computed curvature for the 24 surfaces. We see that, for all contact angles, the error became larger as the grid size became larger. This was because for a low resolution, the number of labeled grid voxels within the pore space was not sufficient to accurately capture the shape of the interface. We could improve the match to the analytical values by changing the number of smoothing iterations for different grid resolutions, however, it is not our purpose to optimize the level of smoothing depending on grid resolutions because a variety of feature sizes can exist in different pore sizes for an application to complex porous media.

The curvature computation method we used was essentially similar to that in Li et al. [11] except for the surface smoothing method: they used constrained Gaussian smoothing, while we used Laplacian smoothing. We also tested constrained Gaussian smoothing for these cases; however, it was found that constrained Gaussian smoothing was inadequate resulting in larger errors when the curvature was small (contact angles close to 90${}^{\circ }$). We refer to Akai et al. [32] for a further detailed comparison of smoothing methods. Furthermore, Li et al. [11] used an average weighted by the distance from the solid to obtain a representative curvature value for a patch of the interface. However, this was not appropriate for our cases—computed curvatures away from the solid wall do not necessarily give an accurate estimation as shown in Figure 9. This was also concluded from our other test cases reported in Akai et al. [32].

In conclusion, for this pore structure with an inscribed radius of 18 μm, most points for a grid resolution finer than or equal to 3 μm fell in the range of ±30% error. In other words, a voxel size 6 times smaller than or equal to the inscribed radius of the pore space provides curvature estimations within ±30% error.

### 3.3. Curvature of the Oil/Water Interface in Complex Pore Spaces

#### 3.3.1. Simulation Conditions

Synthetic bead pack images and micro-CT images of a Bentheimer sandstone were used in this section to study the interfacial curvature in complex pore spaces. These images had a resolution of 3.5 μm/voxel. Pore structures with a size of 256 × 256 × 256 voxels were cropped from the original images and used for the simulations. The porosity of these pore structures were 36% for the bead pack and 19% for the Bentheimer sandstone. For the analysis of the simulation results, the pore structures were divided into pore regions using commercial image analysis software, resulting in 426 and 279 pore regions for the bead pack and Bentheimer sandstone, respectively. The pore size was defined as the radius of the largest sphere that could fit in each pore region. The mean pore radius which accounts for 50% of the pore volume was 37 μm and 30 μm for the bead pack and Bentheimer sandstone, respectively. Hence, the grid resolution was approximately 10 times smaller than the mean pore radius. In Section 3.2, we showed that a grid resolution approximately 6 times the inscribed radius of a channel provided answers accurate to within 30%: we expect in this case, therefore, that the errors will be comparable or smaller.

**Figure 11:**
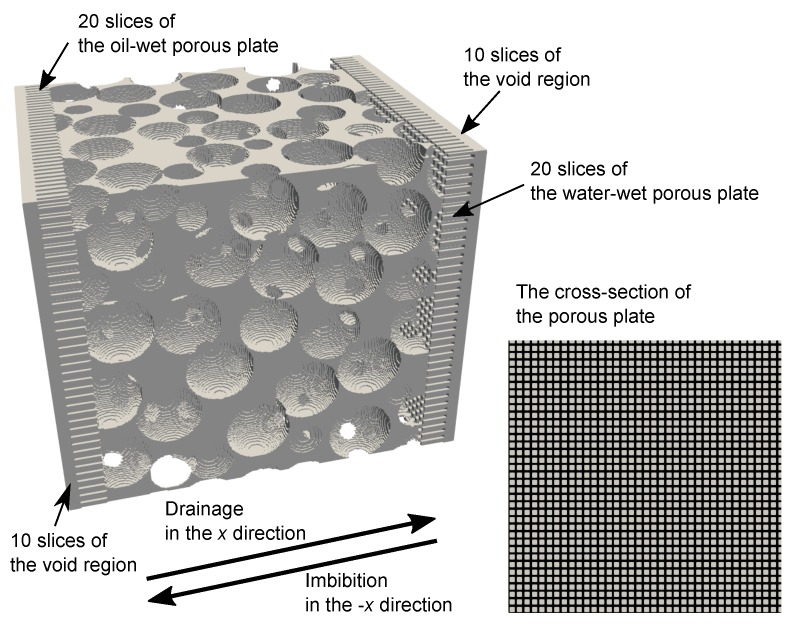
The simulation model used for the study. (**Left**) the pore structure of the bead pack. Here, pore space is shown in white, while solid is transparent. (**Right**) the cross-section of the porous plate attached to the porous domain. Here, pore space is shown in white, while solid is black. This porous plate consisted of the mesh of square pores 5 voxels in width and 20 voxels in length with a contact angle of 150${}^{\circ }$ for the oil-wet porous plate and 30${}^{\circ }$ for the water-wet porous plate.

Similar to a laboratory porous plate capillary pressure measurement, oil-wet and water-wet porous plates were attached to the −x and *x* faces of the porous domain, respectively. This porous plate consisted of a mesh of square pores 5 voxels in width and 20 voxels in length with a contact angle of 150${}^{\circ }$ for the oil-wet porous plate and 30${}^{\circ }$ for the water-wet porous plate. These porous plate domains were followed by 10 slices of a complete void space as a buffer region. Therefore, the simulation domain consisted of 316 × 256 × 256 voxels (Figure 11). Identical density and viscosity of 1000 kg/m${}^{3}$ and 1 mPa were used for the oil and water phases. The contact angle of the pore structure was set to 45${}^{\circ }$ everywhere. The interfacial tension between water and oil was 25 mN/m.

Drainage simulations were performed in the +x direction. Initially, the upstream void space domain was filled with oil, while the rest of pore space was filled with water. During the drainage simulation, the pressure of oil at the upstream boundary ($P_{o}^{+}$) and the pressure of water at the downstream boundary ($P_{w}^{-}$) were controlled by constant pressure boundary conditions. Thus, the drainage simulation was performed by imposing a constant macroscopic capillary pressure ($P_{c}^{M}$), which is given by: $P_{c}^{M}=P_{o}^{+}-P_{w}^{-}$. The macroscopic capillary pressure applied was 1500 Pa and 2500 Pa for the bead pack and Bentheimer, respectively. Since these capillary pressures were below the capillary threshold of the water-wet porous plate, we did not observe breakthrough of oil to the downstream buffer region: the downstream buffer region remained completely filled with water. The simulations were stopped when the capillary number of the displacement became smaller than 1.0 × 10${}^{-6}$.

Imbibition simulations were then performed in the −x direction by reducing the macroscopic capillary pressure. This was performed by reducing $P_{o}^{+}$ while maintaining the same $P_{w}^{-}$ as assigned for the drainage simulations. For both the bead pack and Bentheimer, the macroscopic capillary pressure was reduced to 1000 Pa. The imbibition simulations were also stopped when the capillary number reached 1.0 × 10${}^{-6}$.

#### 3.3.2. Curvature of the Interface

**Figure 12:**
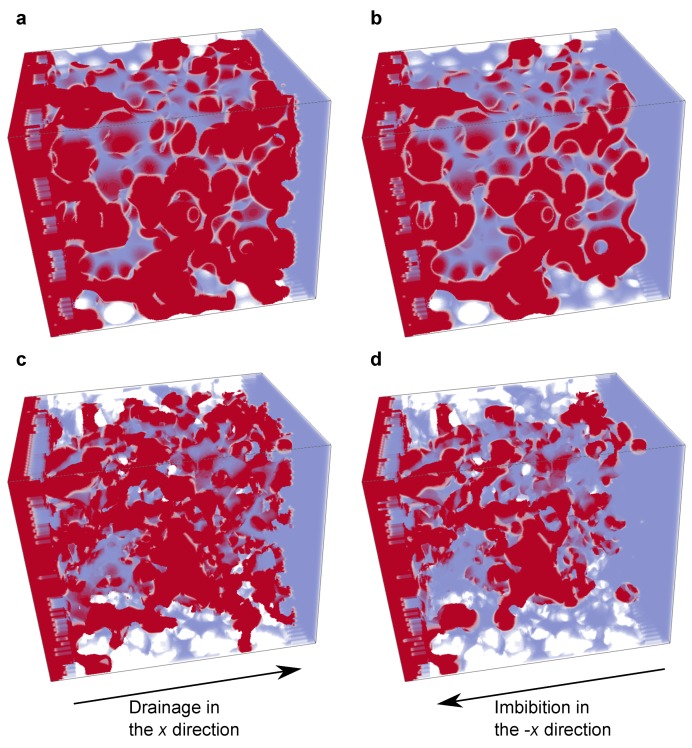
Fluid configurations at the end of (**a**) the drainage in the bead pack, (**b**) imibibition in the bead pack, (**c**) drainage in the Bentheimer sandstone and (**d**) imibibition in the Bentheimer sandstone. Here, oil and water are shown in red and transparent blue, respectively. The drainage simulations were performed from left to right, while the imbibition simulations were performed from right to left.

Figure 12 shows the fluid configurations at the end of the drainage and imbibition simulations. All the analyses presented below were performed only on the pore structure domain; the upstream and downstream buffer regions and porous plates were excluded. Table 2 summarizes the oil saturation and average capillary pressure at the end of the drainage and imbibition simulations, obtained from the computed phase pressures using Equation (15). Note that the capillary pressure shown here was not consistent with the macroscopic capillary pressure imposed. This was partly because the highest capillary pressures locally were observed for interfaces in the oil-wet porous plate region which was excluded from the analysis and partly because the simulations terminated at a capillary number of 1.0 × 10${}^{-6}$ were still in a transient regime and had not yet reached capillary equilibrium.

**Figure 13:**
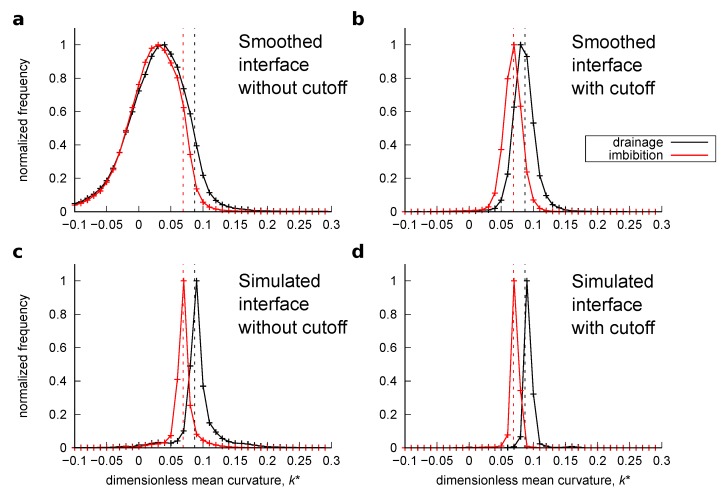
Histograms of the computed curvatures for the bead pack. (**a**) Interfacial curvature computed on the smoothed interface after drainage and imbibition without the distance cutoff and (**b**) with the distance cutoff. (**c**) Interfacial curvature computed on the simulated interface after the drainage and imbibition without the distance cutoff and (**d**) with the distance cutoff.

**Figure 14:**
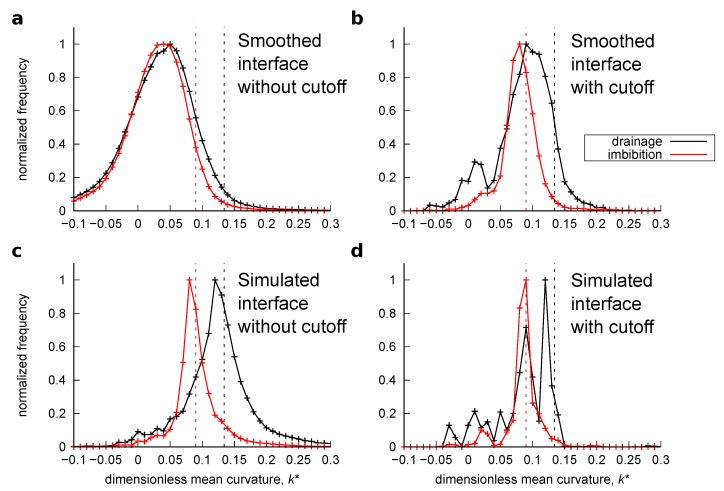
Histograms of the computed curvatures for the Bentheimer sandstone. (**a**) Interfacial curvature computed on the smoothed interface after drainage and imbibition without the distance cutoff and (**b**) with the distance cutoff. (**c**) Interfacial curvature computed on the simulated interface after the drainage and imbibition without the distance cutoff and (**d**) with the distance cutoff.

Similar to Section 3.2.2, the oil/water interface was obtained from the simulation results in two ways: the simulated and smoothed interface. For the smoothed interfaces, Laplacian smoothing with 600 iterations was applied. Curvature was computed on these surfaces. Figure 13 and Figure 14 show the histogram of the computed local curvatures of the drainage and imbibition simulations for the bead pack and Bentheimer sandstone, respectively. Here, both the computed curvatures without the distance cutoff and with 3 voxels of distance cutoff are shown. The average curvature was also obtained from the simulated fluid pressure using Equations (6) and (15) as indicated by vertical dotted lines in the figures.

Here, we can make the following three observations. First, the histograms obtained from the smoothed interfaces without the distance cutoff showed a wide distribution with many negative mean curvature values which was similar to the distribution presented in Herring et al. [12], whereas the histogram obtained from the simulated interfaces showed a narrower distribution with few negative values. This indicates that the overall distribution of the curvatures computed on the smoothed interfaces was distorted by erroneous values, while this was not the case for the simulated interfaces. Second, when the 3 voxel distance cutoff was applied, the histograms computed on the smoothed interfaces became similar to those computed on the simulated interfaces. This suggests that by removing the erroneous values appearing close to three-phase contact lines, curvatures can be properly estimated with the smoothed interfaces. Third, however, the peak curvature values of these histograms were shifted to smaller values. This is evident for drainage in Bentheimer sandstone for both the smoothed and simulated interfaces. This was because the application of the distance cutoff removed data points not just at the edges of large surface patches but also the entire surface in small pores whose radii were smaller than 3 voxels where there were high interfacial curvatures.

**Figure 15:**
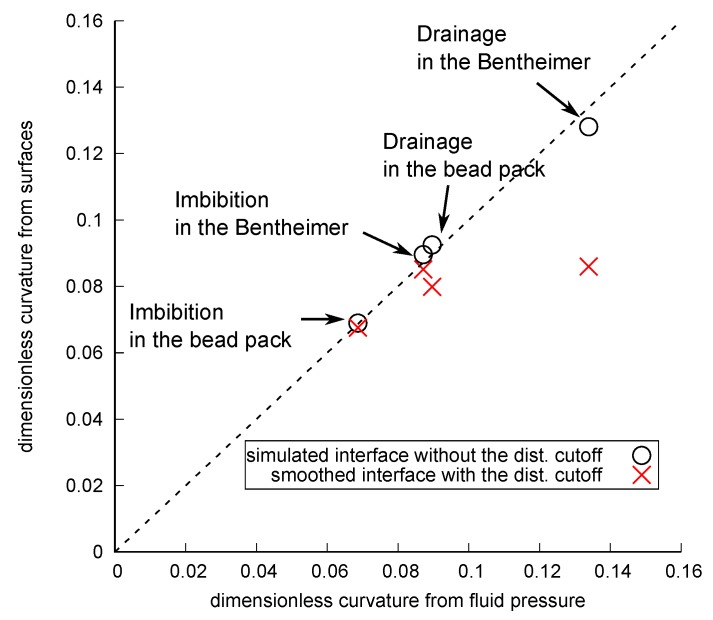
Comparison between the curvature obtained from the average capillary pressure in the porous domain and that obtained from the average curvatures on fluid interfaces after drainage and imbibition for the bead pack and Bentheimer sandstone. The circles show the average value of the computed curvatures on the simulated interfaces without the distance cutoff (see Figure 13b and Figure 14b), while crosses show that computed on the smoothed interface with the distance cutoff (see Figure 13a and Figure 14c).

Figure 15 shows the comparison between the average curvatures obtained from the average fluid pressure in the porous domain and that obtained from the average value of the computed curvatures on the smoothed interfaces with the distance cutoff (Figure 13b and Figure 14b) and the simulated interfaces without the distance cutoff (Figure 13c and Figure 14c) . As discussed above, because the distance cutoff had to be applied to the smoothed interfaces to remove erroneous values, for drainage in the Bentheimer sandstone, the curvature was 36% lower than that found from the fluid pressures, which, as discussed previously, should be close to the correct value. For the other three cases whose dimensionless curvature was less than 0.1 voxel${}^{-1}$, the computed curvatures on the smoothed interfaces were within 11% of the values found from the average pressure. In this specific case at a resolution of 3.5 μm/voxel, a capillary pressure up to 1400 Pa can be estimated within 11% error (see Equation (6)). For the simulated interface, for which the distance cutoff was not necessary, the difference was less than 4% in all cases.

To further investigate the accuracy of the interfacial curvature estimates, curvature was measured on a pore-by-pore basis. First, we obtained the capillary pressure by applying Equation (15) for each pore region. Equation (6) was then used to find the mean curvature. Next, we found the average of computed curvatures on both the smoothed and simulated interfaces for each pore region.

**Figure 16:**
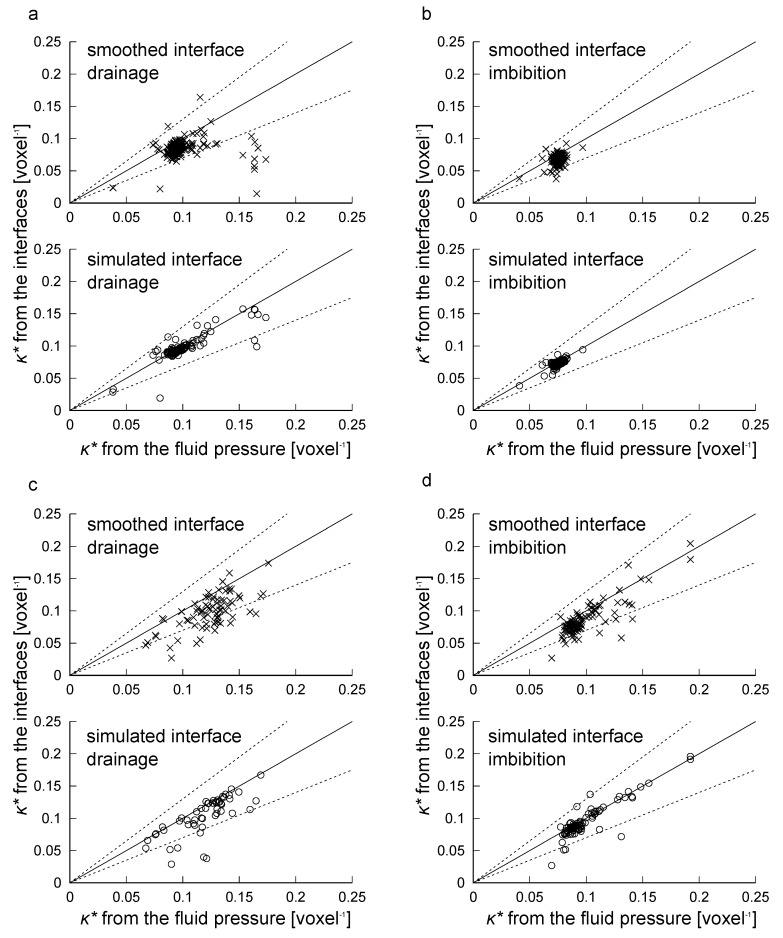
Comparison between the dimensionless mean curvature ($\kappa^{*}$) obtained from the simulated fluid pressure and that obtained from the fluid interfaces. (**a**) Bead pack after drainage; (**b**) Bead pack after imbibition; (**c**) Bentheimer sandstone after drainage; (**d**) Bentheimer sandstone after imbibition. Here, a unit slope indicating perfect agreement in these values is shown by a black solid line, while ±30% difference is shown by the black dotted lines.

Figure 16 compares the dimensionless curvature obtained from fluid pressure and computed values on the interfaces after applying the 3 voxel distance cutoff. For both the drainage and imbibition on the bead pack and Bentheimer sandstone, computed curvatures on the simulated interfaces showed good agreement with those derived from fluid pressure as shown in the lower figures of Figure 16a–d. Although the curvatures on the smoothed interfaces showed larger deviation from the values obtained with fluid pressure, curvatures in most pore regions less than 0.15 voxel${}^{-1}$ fell within ±30% of the pressure-driven values as shown in the upper figures of Figure 16a–d.

The range of dimensionless curvatures for each pore region shown in Figure 16 indicates the variations of local capillary pressure. Hence, as distinguished from previous studies [6,11,12] which used comparisons between a curvature value obtained from transducer-based macroscopic capillary pressure and a mean or peak value of computed curvatures, the range of our values shown in Figure 13 and Figure 14 is supported by the good agreement between the computed curvatures and those obtained from fluid pressure on a pore-by-pore basis.

## 4. Conclusions

To conclude, our suggested curvature measurement method and its likely error can be summarized as follows: (1) imaging should be performed with a voxel size that is at least 6 times smaller than the average pore radius; (2) application of 600 iterations of Laplacian smoothing on the extracted interface with 3 voxels of distance cutoff estimates curvature within a single pore within ±30% error; (3) when this method is applied to complex porous media in which variety of pore sizes exists, local curvatures whose values are less than 0.15 voxel${}^{-1}$ can be estimated within ±30% error; (4) local capillary pressures for each pore can also be estimated within the same degree of error; (5) the average capillary pressure can be estimated within ±11% error if the voxel size is at least 10 times smaller than the average radius of curvature (a dimensionless curvature of 0.1 voxel${}^{-1}$).

Finally, because the simulated interface gave consistent curvatures for each pore region to those from fluid pressure, direct numerical simulations and interfaces extracted from the simulations with sub-resolution smoothness are a useful tool to investigate complex morphology of the interface during immiscible fluid displacement [15,17,18].

## Figures and Tables

**Table 1:** Comparison of capillary pressure between analytical and simulated values. The expressions of analytical capillary pressure for a square tube are provided in Øren et al. [35] for when wetting layers were present in the corners ($\theta <45^{\circ }$) and in Blunt [4] for when wetting layers were absent ($\theta \geq 45^{\circ }$).

Contact Angle [Degrees]	Capillary Pressure		Relative Error
	Analytical [−]	Simulated [−]	
0	1.05$\;\times \;10^{-2}$	1.11$\;\times \;10^{-2}$	5.6%
15	1.03$\;\times \;10^{-2}$	1.05$\;\times \;10^{-2}$	2.7%
30	9.52$\;\times \;10^{-3}$	9.55$\;\times \;10^{-3}$	0.3%
45	7.86$\;\times \;10^{-3}$	7.78$\;\times \;10^{-3}$	−1.0%
60	5.56$\;\times \;10^{-3}$	5.51$\;\times \;10^{-3}$	−0.9%
75	2.88$\;\times \;10^{-3}$	2.86$\;\times \;10^{-3}$	−0.6%

**Table 2:** Summary of the simulation results after the drainage and imbibition simulations. These values were obtained only for the pore structure domain.

	Bead Pack		Bentheimer Sandstone	
	$S_{o}$	$P_{c}$	$S_{o}$	$P_{c}$
	%	Pa	%	Pa
After drainage	83%	1353	71%	2002
After imbibition	71%	1047	51%	1322

## Appendix A. Analytical Expression of a Sphere and Catenoid Surface

Using the parameters *u* and *v*, the surface of a sphere, $S^{S}\left(u,v\right)$, and a catenoid, $S^{C}\left(u,v\right)$, are given by:

(A1) $$\begin{array}{c} S^{S}\left(u,v\right)=\left(\begin{array}{c} x\\ y\\ z \end{array}\right)=\left(\begin{array}{c} c\cos v\cos u\\ c\cos v\sin u\\ c\sin v \end{array}\right),\;S^{C}\left(u,v\right)=\left(\begin{array}{c} x\\ y\\ z \end{array}\right)=\left(\begin{array}{c} c\cosh(\frac{v}{c})\cos u\\ c\cosh(\frac{v}{c})\sin u\\ v \end{array}\right), \end{array}$$

where the superscripts *S* and *C* indicate a surface for a sphere and catenoid, respectively; *c* is a constant which was set to 1 in this study. We consider $\left(u,v\right)\in \left[0,2\pi \right]\times \left[-\frac{1}{2}\pi ,\frac{1}{2}\pi \right]$. The principal curvatures, $\kappa_{1}$ and $\kappa_{2}$, of the these surfaces are written as:

(A2) $$\begin{array}{c} \begin{array}{c} \kappa_{1}^{S}=\\ \kappa_{2}^{S}= \end{array}\begin{array}{c} 1\\ 1 \end{array},\;\begin{array}{c} \kappa_{1}^{C}=\\ \kappa_{2}^{C}= \end{array}\begin{array}{c} {\mathrm{sech}}^{2}v\\ -{\mathrm{sech}}^{2}v \end{array}. \end{array}$$

Therefore, the mean curvature, $\kappa_{m}=\frac{1}{2}\left(\kappa_{1}+\kappa_{2}\right)$, and Gaussian curvature, $\kappa_{G}=\kappa_{1}\kappa_{2}$, are given by:

(A3) $$\begin{array}{c} \begin{array}{c} \kappa_{m}^{S}=\\ \kappa_{G}^{S}= \end{array}\begin{array}{c} 1\\ 1 \end{array},\;\begin{array}{c} \kappa_{m}^{C}=\\ \kappa_{G}^{C}= \end{array}\begin{array}{c} 0\\ -{\mathrm{sech}}^{4}v \end{array}. \end{array}$$

Using these equations, the first to third-order Minkowski functionals can be obtained as:

(A4) $$\begin{array}{ccc} M_{1}^{S} & = & \int dS=2\pi {\left[\sin v\right]}_{v_{1}}^{v_{2}}\;\\ M_{2}^{S} & = & \int \kappa_{m}dS=M_{1}^{S}=2\pi {\left[\sin v\right]}_{v_{1}}^{v_{2}}\\ M_{3}^{S} & = & \int \kappa_{G}dS=M_{1}^{S}=2\pi {\left[\sin v\right]}_{v_{1}}^{v_{2}}\;, \end{array}$$

for the sphere and

(A5) $$\begin{array}{ccc} M_{1}^{C} & = & \int dS=2\pi {\left[\frac{\sinh(2v)+2v}{4}\right]}_{v_{1}}^{v_{2}}\;\\ M_{2}^{C} & = & \int \kappa_{m}dS=0\\ M_{3}^{C} & = & \int \kappa_{G}dS=-2\pi {\left[\tanh(v)\right]}_{v_{1}}^{v_{2}}, \end{array}$$

for the catenoid. Here, the surface integral is performed for the range of u∈[0,2π] and $v\in [v_{1},v_{2}]$, where $v_{1}$ and $v_{2}$ are an arbitrary number which satisfies $-1/2\pi \leq v_{1}\leq v_{2}\leq 1/2\pi$ and are related to the integration interval in the *z*-direction through Equation (A1). For the analysis provided in Figure 6, $v_{1}$ and $v_{2}$ were set to −1 and 1, respectively.
